# Microbiome in cancer: An exploration of carcinogenesis, immune responses and immunotherapy

**DOI:** 10.3389/fimmu.2022.877939

**Published:** 2022-08-08

**Authors:** Pei Zhou, Yawen Hu, Xiaoyan Wang, Luxuan Shen, Xinghao Liao, Yajuan Zhu, Jiadong Yu, Fulei Zhao, Yi Zhou, Hengshui Shen, Jiong Li

**Affiliations:** ^1^ State Key Laboratory of Biotherapy and Cancer Center, Collaborative Innovation Center for Biotherapy, West China Hospital, West China Medical School, Sichuan University, Chengdu, China; ^2^ College of Polymer Science and Engineering, Sichuan University, Chengdu, China; ^3^ Department of Medical Examination, Chengdu Seventh People’s Hospital, Chengdu, China; ^4^ Department of Biotherapy and Cancer Center, State Key Laboratory of Biotherapy, West China Hospital, Sichuan University, Chengdu, China; ^5^ Sichuan Aupone Pharmaceutical Co., Ltd, Chengdu, China

**Keywords:** oncomicrobes, microbiota disorders, gut microbiota, immune cells, cancer immunotherapy, metabolism

## Abstract

Cancer is a major disease endangering human health. More and more studies have shown that microorganisms play an extremely important role in the occurrence, development and treatment of tumors. As a very promising tumor treatment strategy, immunotherapy has also been proved to have a great relationship with microorganisms. Here, the authors review the contribution of the microbiota to cancer and the research on its impact on cancer immunotherapy. We also highlight the possible mechanism of their interaction and outlined the potential application of microbiota in tumor immunotherapy.

## Introduction

The microbiota lives on all the epithelial surfaces of the human body, including the skin, respiratory tract, digestive tract, and urogenital tract, and their presence can be detected even within tumors (1, 2). Hundreds of millions of years of evolution have established a lasting relationship between the microbiome and the human body (3). Past studies have shown that the composition of the microbiota in the epithelial barrier affects systemic functions, including metabolism, nervous system, inflammation, and immunity (4–6). The intestine is the largest digestive organ in the human body. It is constantly exposed to foreign antigens and other environmental factors (7). Microorganisms are distributed along the intestine, and the number of microorganisms in the colon is the largest (8). Studies have found that the imbalance in the intestinal flora is related to the occurrence of many diseases, such as obesity, inflammatory bowel disease, autism and cancers (9). In addition to being closely related to cancer, the microbiome is closely related to the immune system, including innate and acquired immunity (10). Furthermore, many research results show that interventions targeting microbiota, especially intestinal microbes, have achieved gratifying results in cancer immunotherapy (11, 12).

It is worth noting that the relationship between microorganisms and cancer is complex, and there is also an intricate network of factors causing tumor immunotherapy effect by microorganisms (13). Here we review the relationship between carcinogenic microbial infections, microbial disorders, and carcinogenesis. The mechanism of the interaction between microorganisms and immune cells is discussed. We also review the latest reports on the impact of microorganisms on cancer immunotherapy, and finally outlined a new direction for improving the effect of tumor immunotherapy; that is an application of microorganisms.

## Carcinogenic microorganism infections, microbial disorders, and carcinogenesis

### Bacterial and viral infections

Although many microorganisms reside in the human epithelial barrier, only 12 (1 bacteria, 8 viruses and 3 parasites) are currently considered human carcinogens by the International Agency for Research on Cancer (IACR) (14). The most well-known microorganisms associated with cancer is *Helicobacter pylori* (*H. pylori*), which is considered the most common pathogen of infection-related cancers (15). *H. pylori* is a gram-negative bacterium that can selectively colonize the gastric epithelium (16). About half of the world’s population is infected by *H. pylori (* 17). Colonization with it causes no symptoms in humans; however, long-term colonization with *H. pylori* significantly increases the risk of gastric cancer (18, 19). The carcinogenic virulence factors of gastric cancer are closely related to *H. pylori* virulence proteins such as CagA, VacA, and CagPAI (20, 21). Besides the virulence factors, *H. pylori*-induced oxidative stress, DNA damage, up-regulation of pro-inflammatory cytokines, and activation of multiple signaling pathways are all responsible for *H. pylori*-induced gastric cancer (22). These have been discussed in several articles (22–24). Notably, while *H. pylori* is the only bacterium currently considered to be a human carcinogen, many other bacteria have also been reported to be closely linked with cancer development. Studies have shown that pks^+^ strains of *Escherichia coli* (*E. coli*) can synthesize colibactin to induce DNA double-strand breaks (25). Besides, colibactin can not only induce the emergence of senescent cells, which promote tumor growth by the secretion of growth factors, but also change the immune microenvironment through impairment of antitumor T-cell response, leading to tumoral resistance to immunotherapy (26, 27). With the development of organoid technology, the mutation characteristics of colon cancer caused by colibactin are being gradually clarified (25, 28, 29). Enterotoxigenic *Bacteroides fragilis* toxin cleaves E-cadherin, resulting in Wnt/β-catenin signaling and alter the gene expression in colonic epithelial cells (14, 25, 30). These factors are all potential factors leading to colorectal cancer (CRC) (31). In addition, *Propionibacterium acnes* (*P. acnes*) stimulate prostate cells to secrete interleukin (IL)-6 and IL-8, which may be related to the occurrence and development of prostate cancer (32).

The mechanisms by which tumor viruses promote carcinogenesis are more diverse. For example, human papillomavirus (HPV), Epstein-Barr virus (EBV), and Merkel cell polyomavirus (MCPyV) cause tumors by encoding oncogenic proteins that can regulate cell proliferation, apoptosis, or blood vessels generated to promote the occurrence of cancer (33). Other human tumor viruses, such as hepatitis B virus (HBV) and hepatitis C virus (HCV), do not express definitive oncogenic proteins, but cause tumorigenesis primarily by inducing a chronic inflammatory state (34, 35). Simultaneously, sustained inflammatory and immune responses can lead to increased production of reactive oxygen species (ROS) and reactive nitrogen species (RNS); thus inducing gene mutations (36). The promotion of genomic instability by these factors is one of the mechanisms by which viral infection promotes cancer development.

### Microbiota dysbiosis

Improvement in socioeconomic factors is often associated with detrimental lifestyle changes and environmental exposures that are major determinants of cancer (37). An interesting study shows that for all cancers and a large number of cancer types, there is a strong and positive correlation between cancer incidence and national socioeconomic level in both men and women (37). There is a growing recognition of another gene pool, the microbiome, that needs to be considered when assessing the impact of environmental factors on human health (38). Environmental and host-related factors can drive dysbiosis, which is defined as changes in the composition and function of the microbiota (39). There is increasing evidence that dysbiosis of the microbiota is associated with cancer development, and this relationship is particularly evident with the gut microbiota. Human studies have shown that compared with healthy individuals, patients with CRC have a less diverse gut microbiome (40). Furthermore, at different CRC stages, ranging from adenomatous polyps to early-stage cancer to metastatic disease, the microbiome undergoes specific changes, including a marked increase in the DNA and RNA levels of *Fusobacterium nucleatum* (*F. nucleatum*) in human CRC (40–42). In addition, the gut microbiota also affects normal intestinal stem cells (ISCs). Dysbiosis induces aberrant programming of ISCs through multiple mechanisms, leading to the transformation of ISCs into cancer stem cells, which are thought to initiate CRC (43). Dysregulation of gut microbes not only affects the occurrence and development of CRC locally, but also affects the occurrence of distant organ cancers. The majority of liver cancers occur in patients with cirrhosis, and these patients often exhibit leaky gut and dysbiosis, which are thought to be the main cause of liver cancer in patients with cirrhosis (44). Patients with hepatocellular carcinoma (HCC) have been reported to have higher levels of *E. coli* and other Gram-negative bacteria in the gut microbiota compared with healthy individuals (45). On the other hand, gut microbes in patients with HCC have reduced levels of *Lactobacillus* spp., *Bifidobacterium* spp., and *Enterococcus* spp (46).. A new study shows that gut microbes can even influence the production of male hormones to interfere with the development of prostate cancer in castrated mice (47). In addition to the gut microbiota, dysbiosis of other epithelium-distributed microbes is also closely linked to carcinogenesis at their colonization sites. *Lactobacillus* is the dominant genus of human vaginal microbiota at reproductive age (48). The cervicovaginal microbiota is dominated by *Lactobacillus crispatus, Lactobacillus iners, Lactobacillus gasseri*, or *Lactobacillus jensenii*, which help to maintain the pH of the vagina (49). Studies have reported that women with or at risk of developing ovarian cancer have an imbalance in the cervicovaginal microbiota, as manifested by a reduced ratio of *Lactobacillus* to total vaginal microbes (50).

The causes of microbe disorders in the human body are diverse, including diet, antibiotics, genetics, family transmission, and other factors (39). Here, we mainly discuss the impact of infection and inflammation on dysbiosis. As mentioned earlier, *H. pylori* is the strongest risk factor identified for gastric cancer, and studies have shown that *H. pylori*-negative individuals have a highly diverse gastric microbiome (51). When 1833 bacterial clones from 23 gastric biopsy samples were analyzed, sequencing identified 128 phylotypes within 8 bacterial phyla. In contrast, only 33 phylotypes were detected in three *H. pylori*-infected populations (52). These data suggest that *H. pylori* colonization greatly reduces the overall diversity of the gastric microbiota. However, in many studies, the composition of microorganisms varies greatly between individuals, and the mechanism of how *H. pylori* colonization affects other microorganisms in the stomach still needs to be further explored. *F. nucleatum*, an invasive and pro-inflammatory bacterium known to cause oral and gastrointestinal infections, has also been detected in tumors from CRC patients (53–56). *F. nucleatum* is a potent stimulator of the inflammatory cytokines, IL-6, IL-8, and TNF-α, and regarding dysbiosis, *F. nucleatum* induces increased inflammation, becoming a pathogen (57). In CRC, *F. nucleatum* not only activates the inflammatory response but also promotes colorectal carcinogenesis through its FadA adhesin regulation of E-cadherin/β-catenin signaling (58). Its regulation of autophagy also promotes chemoresistance in CRC (59).

### Intratumoral and local tumor microbes

With advances in sequencing technology, the presence of microbes within tumors has been identified. Studies show that each cancer subtype has a unique microbiome with specific metabolic functions, and the intratumor bacteria are mostly present in both cancer and immune cells (2, 60). Therefore, the intratumoral microbiome plays a crucial role in tumor development and treatment. Above, we mentioned various microorganisms that are in direct contact with gastrointestinal tumors, such as *H. pylori*, pks^+^ *E. coli*, and *F. nucleatum*, which are involved in the occurrence and development of gastric or CRC. Therefore, here we mainly focus on intratumoral and local tumor microbes in other tumors. In a spontaneous murine mammary tumor model, Fu et al. (61) found that the depletion of intratumoral bacteria *via* tail vein injection of mixed antibiotics that had no effect on the gut microbiota, significantly reduced lung metastasis without affecting primary tumor growth. Shi et al. (62) found that *Bifidobacterium* in the gut can accumulate in the tumor microenvironment, and intratumoral injection of very low doses of mixed antibiotics reduced the efficacy of anti-CD47 immunotherapy in tumor-bearing mice. Boesch et al. (63) found compared to healthy lung tissue that the lung tissue of patients with non-small cell lung cancer had a higher abundance of *Gammaproteobacteria*, which correlates with low programmed death-ligand 1 (PD-L1) expression and worse overall survival (OS) under immune checkpoint inhibitor (ICI) therapy. Ma et al. (64) analyzed microbial compositions of intratumor bacteria in prostate cancer to determine the influence of the microbiome on metastatic growth. They identified specific microbes that can significantly deter the development of prostate cancer (*Listeria monocytogenes* and *Methylobacterium radiotolerans JCM 2831*) or contribute to cancer aggressiveness (*Stackebrandtia nassauensis DSM 44728 and Mycoplasma hyorhinis HUB-1*). In terms of the mechanisms by which intratumoral microorganisms affect tumor progression, DNA damage, immunosuppression, drug metabolism, and activation of oncogenic pathways are still the main mechanisms (65). Recently, an interesting study showed that fungi within mouse pancreatic ductal adenocarcinoma (PDAC) tissue can drive IL-33 secretion, further recruit and activate T_H_2 cells and innate lymphoid cells 2 (ILC2) in tumor issue, ultimately leading to the inhibition of anti-tumor immune response and promotion of tumor progression (66). Accordingly, intratumoral and local tumor microbes remain a promising research direction for tumor progression.

**Figure 1:**
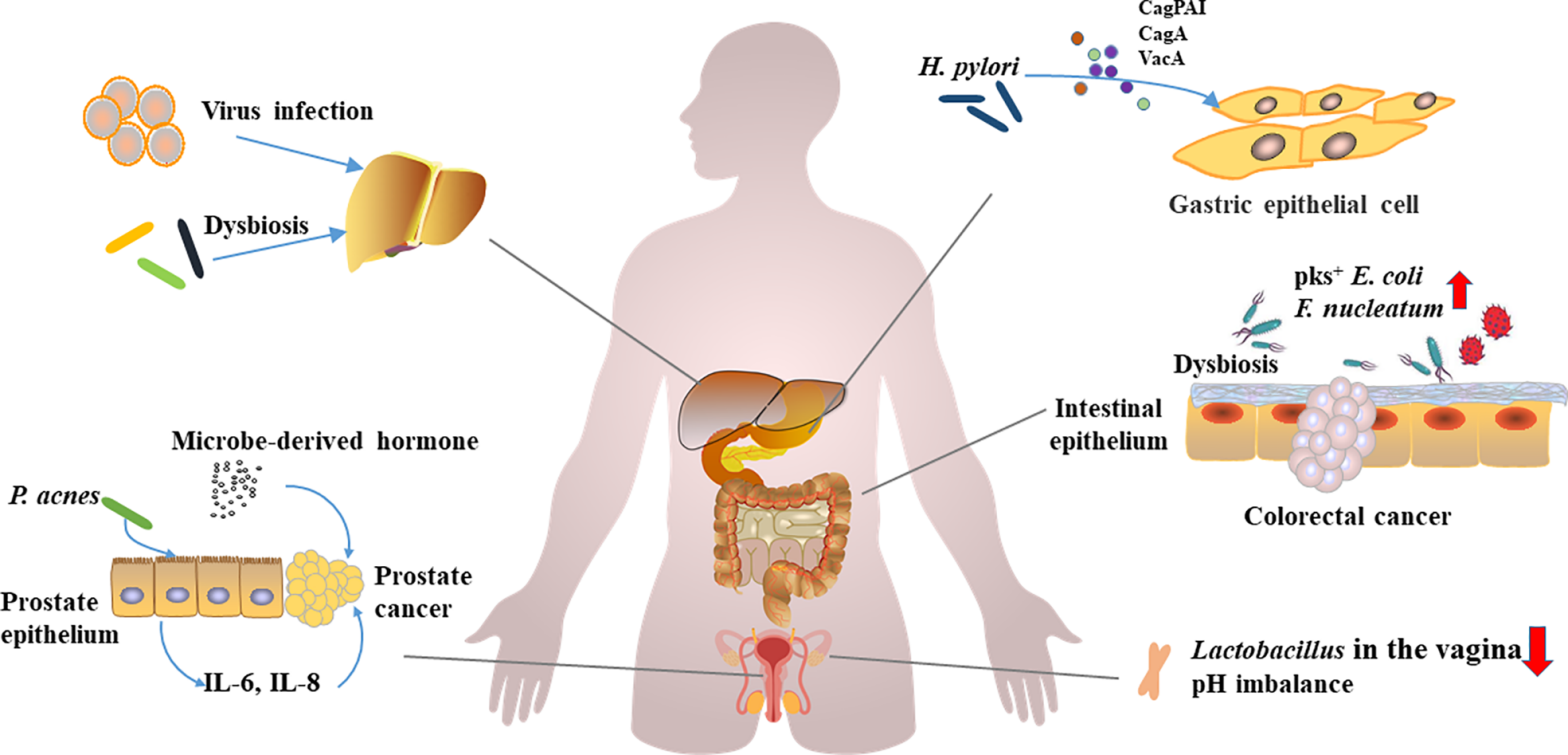
Carcinogenic microorganism infections, microbial disorders, and carcinogenesis.Viruses, oncogenic microorganisms infections, and dysbiosis of the microbiota have been implicated in the development of multiorgan cancers. *H. pylori* promotes gastric carcinogenesis through virulence factors (CagPAI, CagA, and VacA etc.). *F. nucleatum* and pks^+^ *E. coli* can promote the development of colorectal cancer. *Lactobacillus*, a vagina-dominant genus that helps regulate pH, is reduced in abundance in ovarian cancer patients. *P. acnes* induces prostate cancer by stimulating the production of IL-6 and IL-8 in prostate cells, and hormones derived from microorganisms promote the development of prostate cancer. Hepatitis virus induces hepatitis, and persistent inflammation and dysbiosis can affect the occurrence of liver cancer.

In general, carcinogenic microbial infection and dysbiosis of microbiota are closely related to tumorigenesis (**Figure 1**), and intratumoral and local tumor microbes also have a significant contribution to tumor progression. However, the causal relationship among them still needs to be further studied.

## Microbiota and cancer immunotherapy

Cancer immunotherapy is an approach that harnesses the immune system to fight cancer (67). Current immunotherapy can be roughly divided into oncolytic virus therapies, cancer vaccines, cytokine therapies, adoptive cell transfer (ACT), and ICIs (68). Their common features are enhanced immune responses, including innate immunity and/or adaptive immunity to clear cancer cells. The microbiota and its metabolites provide key signals for the induction, development, and function of the host immune system (69, 70). Growing evidence suggests that the microbiome plays a key role in cancer immunotherapy, and here we link the microbiome and immunotherapy from three perspectives: adaptive immunity, innate immunity, and metabolism.

### Linking microbiota and cancer immunotherapy from adaptive immunity perspective

Cytotoxic T lymphocyte-associated antigen 4 (CTLA-4), programmed cell death protein 1 (PD-1) and its ligand (PD-L1) are important immune checkpoints and are also important factors in regulating T cell immune function (71). Inhibition of these targets reactivates T cells more effectively, provides novel treatments for a variety of cancers including melanoma, non-small cell lung cancer (NSCLC), and significantly improves patients survival (72). However, these therapies targeting immune checkpoints are not effective in all patients. Pembrolizumab, an anti-PD-1 monoclonal antibody, has been shown in clinical studies to respond better in patients with lung metastases compared to patients with liver metastases (62% vs. 22%) (73). Anti-CTLA-4 blockade using ipilimumab is the first treatment to prolong OS in patients with advanced melanoma in a randomized setting (74). In a study of 30 patients with melanoma treated with ipilimumab, only 11 (37%) had their disease under control (75). Therefore, how to improve the patient’s response to monoclonal antibodies has become an important issue. In recent years, increasing number of studies have shown a significant impact of intestinal microbiota on treatment with ICIs.

Gopalakrishnan et al. (76) divided 112 melanoma patients receiving PD-1 immunotherapy into responder and non-responder groups to determine significant differences in the diversity and composition of the gut microbiome between them. *Faecalibacterium* was more abundant in fecal microbiome responders, while fecal microbiome non-responders had higher abundance of *Bacteroides thetaiotaomicron*, *E. coli*, and *Anaerotruncus colihominis*. More CD8^+^ T cells infiltration and stronger systemic antitumor immune responses were observed in the tumors of responders (76). Similar studies revealed that antibiotics inhibited the beneficial effects of ICIs in patients with advanced cancer and that patient response to ICIs was associated with a relative abundance of *Akkermansia muciniphila* (Akk) (12). Oral Akk supplementation restores the response to PD-1 blockade in an IL-12-dependent manner by increasing the recruitment of CCR9^+^ CXCR3^+^ CD4^+^ T lymphocytes in mouse tumor beds (12). Oral administration of *Bifidobacterium* modulates the activation of mouse DC cells, improves the effector function of CD8^+^ T cells, and enhances the efficacy of PD-L1 (77). In studies of CTLA-4, similar results were observed with PD-1. A study found that germ-free mice did not respond to CTLA-4 blockade, the efficacy of CTIA-4 blockade was affected by *B. fragilis, B. thetaiotaomicron*, and *Burkholderiales*, and these microorganisms also affected IL-12-dependent Th1 immune responses (78).

A large proportion of cancer patients do not benefit from ICIs therapy, and of those who do, some responders still relapse after a period of response (79). Absence of a relevant number of immunogenic tumor antigens, defects in the antigen processing and presenting machinery, and insufficient T cell infiltration are all mechanisms of the resistance (80). Interestingly, ICIs therapy seems to be more effective in tumors with a high tumor mutational burden (TMB), and the reason may be related to the neoantigens produced by the tumor (81).The cross-reactivity of T cells allows each T cell to recognize multiple antigens (82). The human microbiome is a huge gene pool, and neoantigens produced by tumors may be mimicked by peptides encoded by the microbiota. When tumor neoantigens appear, memory T cells can quickly provide protection (80, 83). Bessell et al. (84) found that T cells targeting an epitope called SVYRYYGL (SVY), expressed in the commensal bacterium, *Bifidobacterium breve* (*B. breve*), cross-react with a model neoantigen, SIYRYYGL (SIY). Moreover, *B. Breve* colonization can shape SVY- reactive T cell receptor library, influence T cell response, and then affect the growth of tumor that expresses neoantigens (84). Balachandran et al. (85) found that neoantigen-specific immunity gained during primary tumor outgrowth could be associated with decreased relapse and prolonged survival. Taken together, tumor antigen mimicry generated by the microbiota and cross-reactivity of T cells may be beneficial for tumor immunotherapy, and these are possible explanations for the large differences in response to checkpoint inhibitors in cancer patients (86).

### Linking microbiota and cancer immunotherapy from innate immunity perspective

Cytotoxic T lymphocytes (CTL), especially CD8^+^ T lymphocytes, are the main anti-tumor effector cells and the main target cells for tumor immunotherapy (87). However, innate immune cells also play an important role in cancer immunotherapy. Current research shows that innate immunity not only indirectly affects anti-tumor immune responses by controlling T cells, but also directly and critically shapes the tumor microenvironment (85), which is an important part of tumor immunity (88). Crosstalk between the microbiota and innate immunity affects multiple aspects of body homeostasis, and this complex bilateral interaction is critical for human health (89). Therefore, some tumor immunotherapies targeting innate immune cells have been developed, and the influence of the microbiota on innate immune cells has also been confirmed to be relevant to a variety of cancer immunotherapies.

Pattern recognition receptors (PRRs) are an important part of innate immune defense, and they are expressed on a variety of immune cells such as leukocytes and macrophages (90). PRRs respond to a variety of bacterial and viral ligands, also known as pattern-associated molecular pattern (PAMP), including peptidoglycan (PGN), lipopolysaccharide (LPS), double-stranded RNA, and CpG DNA (90, 91). PRRs genes, including *NOD1/2*, *NLRP3*, and various toll-like receptor (TLR) genes, recognize PAMPs as non-self-entities and trigger intracellular signaling pathways that induce a variety of cytokines and chemokines that help maintain host response against infection (91, 92). Gram-negative bacterial cell wall component, LPS, is recognized by TLR4, and activation of TLR4 promotes prostate cancer development and induces nitric oxide and IL-6 production in CRC (93). TLR3 agonist, poly(I:C), was developed to mimic infection by pathogens and boost immune system activation to promote anti-cancer therapy (94). NOD2 receptor is an inflammatory pathway and microbiota modulator, and studies demonstrate that loss of NOD2 activity led to more severe colitis and a higher risk of adenoma and CRC in mouse models (95). In conclusion, the rich innate immune signaling pathways initiated by the microbiota through PRRs are important in infection, inflammation, and cancer development (96).

TME is a complex system that includes many different types of cells, abnormal vasculature, and immunosuppressive cytokines, and it is one of the important reasons why tumors evade immune surveillance (88, 97). Mononuclear phagocytes (MPs) (i.e., monocytes [Mo], macrophages [Macs], and dendritic cells [DCs]) are the major innate immune cells and important components of the TME (98). A recent study sheds light on the effect of the microbiota on MPs in TME and innovatively proposed that MPs in TME can be remodeled by microorganisms to improve ICIs efficacy. Lam et al. (98) demonstrated that microbiota-derived stimulator of interferon gene (STING) agonists such as c-di-AMP induce type I interferon (IFN-I) production by intratumoral Mo, which regulates their skewing and natural killer (NK)-DC crosstalk. The triggering of this mechanism can be achieved by a high-fiber diet, which enriches *Akkermansia muciniphila*, further produces c-di-AMP, and enhances the therapeutic effect of ICIs in melanoma patients. Monocytes are more inclined to differentiate into tumor-promoting Macs when the microbiota is adversely disrupted (98). Another study also found that *Bifidobacterium* colonized in the tumor microenvironment can effectively stimulate STING signaling and increase cross-priming of DCs after anti-CD47 treatment (62). Overall, these studies revealed possible mechanisms of the interaction between the microbiota and innate immune cells, and we believe that more specific mechanisms will be explored in the future.

ACT therapy is an immunotherapy method in which autoimmune cells, especially T and NK cells, are isolated, modified, amplified, and re-injected into a patient to eliminate cancer cells (68, 99). Chimeric antigen receptor-T (CAR-T) cell therapy is used to treat different malignant tumors including lymphoma and leukemia, and it is one of the promising ACT therapies (100). Like ICIs, CAR-T therapy is not effective in all patients. The complete response rate in patients with aggressive lymphoma is from 40% to 60%, and a large proportion of patients will relapse (101). Recent studies have shown that oral administration of vancomycin, an antibiotic mainly targeting Gram-positive bacteria, can improve the efficacy of CAR-T therapy in mice with cervical cancer. Mechanistically, vancomycin treatment induces an increase in systemic CD8α^+^ DCs, which sustains systemic adoptively transferred antitumor T cells in an IL-12–dependent manner (102). NK cells, as the name suggests, are non-specific tumor-killing cells in innate immunity. They do not need any antigenic priming before attacking the target, and can quickly kill tumor cells through a variety of mechanisms (103, 104). Therefore, CAR-NK has some significant advantages over CAR-T, such as multiple mechanisms of activating cytotoxic activity and better safety (103). Although there is no clear report on the association between CAR-NK and the microbiome, existing studies have shown that high-salt diet (HSD) increases the abundance of *Bifidobacteria* and leads to increased intestinal permeability, which further leads to *Bifidobacteria* colonization within tumors, enhancing NK cell function and promoting tumor regression (104). The use of mixed antibiotics has also been found to promote glioma growth in mice, which is associated with disruption of the gut microbiota and reduction of cytotoxic NK cell subsets (105). These evidence give us reason to believe that the microbiota may contribute to CAR-NK therapy.

### Linking microbiota and cancer immunotherapy from metabolism perspective

The gut microbiota can ferment undigested food in the colon and can also utilize endogenous compounds produced by the host (70). Some of the diverse metabolites produced by microorganisms can enter and interact with host cells, thereby affecting immunity and disease risk (106). Multiple metabolites produced by the microbiota have also been shown to be relevant for tumor immunotherapy.

Short-chain fatty acids (SCFAs) are the main end-products of indigestible carbohydrates fermented by gut microbiota, mainly including formate, acetate, propionate, and butyrate (107). Among them, butyrate has been shown to have a potential role in immune regulation, inhibiting nuclear factor activation in macrophages and also inhibiting histone deacetylation in acute myeloid leukemia, while exerting an inhibitory effect on CRC (107, 108). Butyrate and propionate inhibit LPS-induced expression of cytokines such as IL-6 and IL-12p40, exhibiting strong anti-inflammatory effects (109). SCFAs have been recognized to maintain intestinal homeostasis by regulating different cells. SCFAs can enhance mucus production by goblet cells, while promoting the production of IL-22 by CD4^+^ T cells to maintain intestinal epithelial barrier function (110, 111). A growing number of studies have shown that the gut microbiota can influence tumor immunotherapy through SCFAs. A new study shows that valeric acid and butyric acid enhance the antitumor activity of CTL and CAR-T cells through metabolic and epigenetic reprogramming. The mechanism lies in the increased production of effector molecules such as CD25, IFN-γ, and TNF-α (112). Through oral administration of pectin, inulin, and other polysaccharide dietary fibers in mice, researchers have found that they can significantly improve the therapeutic effect of PD-1 mAb. All can increase the relative abundance of key symbiotic microorganisms (such as *Akkermansia* and *Lactobacillus*) and SCFAs, further promoting the invasion of CD8 ^+^ T cells into the tumor (113, 114). In addition, SCFAs were found to increase the memory potential of antigen-primed CD8^+^ T cells and trigger their differentiation into stem cell-like Tcf1^+^PD-1^+^CD8^+^ T cells, resulting in potent and long-term antitumor effects (113, 115). It is worth mentioning that in addition to SCFAs, other lipid metabolisms also have an impact on cancer immunotherapy, such as glycerophospholipid metabolism and sphingolipid metabolism (116). However, studies on the impact of lipid metabolism on tumor immunotherapy still focus on effector T cells (117).

Amino acid metabolism is also an important aspect of host and microbial metabolism, which also plays an important role in cancer immunity. Reacquiring durable immune memory is challenging in the setting of severe T cell exhaustion, and exhausted T cells exhibit distinct histone profiles and limit tumor immunotherapy (118, 119). Studies have found that tumor cells compete with CD8^+^T for methionine through the high expression of methionine transporter, which reduces the levels of methionine and methyl donor s-adenosylmethionine (SAM) in T cells, and inhibition of transporters enhances immune checkpoint-induced tumor immunity (118). Elevating L-arginine levels induces global metabolic changes including a shift from glycolysis to oxidative phosphorylation in activated T cells, promoting the production of central memory-like cells with antitumor activity in mice model (120). Moreover, blocking glutamine metabolism not only inhibits tumor growth, but also enhances the efficacy of ACT and PD-1 mAb. The mechanism involves the blocking of glutamine metabolism, which inhibits glucose metabolism through the tricarboxylic acid cycle and glycolysis-related pathways (121). L-tryptophan contributes much to maintaining the balance between the gut microbiota (122). Changes in the microbiota can also modulate tryptophan and its metabolites (including kynurenine) and thus affect the host immune system (123). Researchers found that ginseng polysaccharides (GPs, a polysaccharide extracted from ginseng) could significantly improve the therapeutic effect of PD-1 mAb in tumor-bearing mice. Mechanically, oral administration of GPs increases valeric acid produced by microbial metabolism and decreases L-kynurenine and Kyn/Trp ratios (124).

Notably, in addition to lipid metabolism and amino acid metabolism, other metabolic pathways of the microorganism and host also appear to have an impact on cancer immunotherapy. *B. pseudolongum*, an intestinal *Lactobacillus* bacterium, enhances immunotherapeutic responses by producing the metabolite, inosine. Specifically, inosine promotes Th1 cell activation in a context-dependent manner through T cell-specific A_2A_ receptor signaling for immune enhancement (125). Purine metabolism, a downstream metabolic pathway of inosine, has also been shown to be involved in host immunity. A recent study demonstrated that priming of the purine nuclease FAMI in DC inhibits CD4^+^ and CD8^+^ T cell priming. DCs lacking FAMIN activity enhance antigen-specific cytotoxicity, IFN-γ secretion, and T cell expansion (126). Rhein can increase *Lactobacillus* levels, alter purine metabolism, and reduce uric acid levels in the gut and further alleviate dextran sulfate sodium (DSS)-induced enteritis in mice (127). These evidence suggest that microbe-mediated purine metabolism seems to have great research prospects in inflammatory to cancer transformation.

## Applications of microorganisms: A new strategy to improve cancer immunotherapy

The impact of microorganisms on immunity is multi-faceted, and microorganisms are increasingly being applied to various immunotherapy to improve immunotherapy. Even the microbes themselves are being used as new targets for immunotherapy.

### Efficacy improvement: Diet, probiotic use, and fecal microbiota transplantation

Diet is a key factor in altering gut microbiota composition and function (128). Appropriate intake of dietary fiber and prebiotics has been recognized as a positive contribution to human health, including weight control, cardiovascular protection, blood sugar control, and brain health (129, 130). Based on the profound impact of the microbiota on tumor immunotherapy, an increasing number of preclinical studies have attempted to improve immunotherapy through dietary interventions. Previous studies have found that oral administration of inulin and pectin can enhance the efficacy of ICIs, which is related to the change in intestinal flora and metabolism (113, 114). We have described a specific mechanism in the last section. Messaoudene et al. (131) gavaged mice with polyphenol-rich berry camu-camu and found that the berry significantly enhanced the efficacy of ICIs. The main active component of this berry, castalagin, alters bile acid metabolism in mice and binds to the surface of *Ruminococcus bromii*, resulting in better antitumor activity of PD-1 antibody. Spencer et al. (132) conducted a high-fiber dietary intervention in patients receiving ICIs and found that higher dietary fiber was associated with significantly improved progression-free survival in patients on ICIs.

Probiotics are defined as live microorganisms that, when ingested in sufficient amounts, confer a health benefit to the host (133). Colonization with probiotics is beneficial to the host in the long run. However, it is a long and arduous process from oral probiotics to colonization of probiotics in the intestine, which is affected by various aspects such as colonization resistance, intestinal mucosa, and mucus layer (134). van Zyl et al. provide a detailed review of the *in vivo* kinetics of multiple probiotics following oral administration. By comparing the number of cells in feces before and after ingesting a particular strain, they found differences in survival and persistence between genera and even between strains (135). However, previous research has focused on the role of probiotics in intestinal diseases, especially intestinal inflammation and diarrhea (135, 136). With the rise in immunotherapy, in recent years, improving immunotherapy through probiotic supplementation has also become an emerging research direction. A new study found that microbial exopolysaccharide produced by *Lactobacillus delbrueckii* subsp. *bulgaricus* OLL1073R-1 (EPS-R1) induced CCR6^+^ CD8^+^ T cells in mice and humans. In mouse models of colon adenocarcinoma and breast cancer, ingestion of EPS-R1 augmented the antitumor effects of anti-CTLA-4 or anti-PD-1 mAb (137). Another study also found that *Clostridioides butyricum* MIYAIRI 588 strain significantly improved OS in patients with NSCLC treated with ICI therapy (138). Colonization of *Bifidobacterium pseudolongum*, *Lactobacillus johnsonii*, and *Olsenella* species in the gut enhances the efficacy of CTLA-4, which is associated with CD4^+^ and CD8^+^ T cell activation (125). High abundance of AKK appears to correlate with better efficacy of ICIs in both humans and mice (12). As a potential star probiotic, AKK has been proven to improve tumor immunotherapy, as well as improve obesity, anti-diabetes, and inhibit inflammation in mice (139). Interestingly, both live and inactivated AKK had positive health implications (140, 141). The adverse reactions of ICIs involve skin, gastrointestinal tract, thyroid, heart, and other organ systems (142). Some microbiota can also reduce the adverse reactions caused by ICIs. For example, *Bifidobacterium* can alleviate colitis induced by ipilimumab (CTLA-4 inhibitor) treatment by inhibiting the release of pro-inflammatory cytokines (143). However, it is worth mentioning that the focus on a certain immunotherapy-enhancing probiotic often occur after the use of ICIs, and precise probiotic supplementation (such as *bulgaricus* OLL1073R-1) is still an aspect that requires attention in basic research and preclinical trials, as well as areas that need to be expanded.

Fecal microbiota transplantation (FMT) is defined as the transplantation of gut microbiota from healthy donors to diseased patients *via* an upper or lower gastrointestinal route to restore gut microbial diversity (144). In human medicine, FMT was originally used to treat microbial-induced gastrointestinal diseases such as *Clostridioides difficile* infection and ulcerative colitis (145–147). FMT is now being used more widely, including for the treatment of metabolic syndrome, diabetes, Crohn’s disease, Parkinson’s disease, multiple sclerosis, psoriasis, anorexia nervosa, or Alzheimer’s disease (148). The impact of FMT on the microbiome has led researchers to see its potential in tumor immunotherapy. Baruch et al. found that combining FMT (from complete response donors) with reinduction anti-PD-1 therapy is safe, feasible, and potentially effective in patients with refractory metastatic melanoma (149).

In addition to ICIs, microbes have a facilitating role in other immunotherapies. Combination of oral Wilms’ tumor 1 (WT1) cancer vaccine and anti-PD-1 antibody treatment using a *Bifidobacterium* vector has been shown to eliminate tumor growth in a syngeneic mouse model of bladder cancer (150). Vaccine delivery based on the antigenic action of the microbiota may significantly inhibit tumor-associated microorganisms, such as *H. pylori*, which possesses a variety of bacterial toxins and proteins, and can serve as key candidates for *H. pylori* vaccine construction (151). Following radiation therapy, intratumoral injection of genetically attenuated *Salmonella* strains coated with antigen-adsorbing cationic polymer nanoparticles resulted in tumor antigen accumulation around the tumor (152). This enhances crosstalk between antigens and DCs, and the use of flagellated bacteria to transport tumor antigens around tumors to enhance DC activation may open up new strategies for *in situ* cancer vaccination (152). In cytokine therapy, beneficial commensal microorganisms, AKK, combined with IL-2, can enhance the antitumor efficacy of IL-2 and enhance immune surveillance. Mechanistically, the antitumor immune response elicited by AKK is partially mediated by Amuc, derived from the outer membrane protein of AKK, through activating TLR2 signaling pathway (153).

### A new target for immunotherapy: The microbiome itself

Microbiota itself holds great promise as a new target for immunotherapy. Montalban-Arques et al. (154) found that four butyrate-producing *Clostridioides* species: *Roseburia gutis, Eubacterium hallii, Faecalibacterium prausnitzii*, and *Anaerostipes caccae* (CC4) can prevent tumor development, including CRC, melanoma, breast, and lung cancers. Specifically, CC4 supplementation increases the frequency and activity of tumor-infiltrating IFN-γ^+^ CD8^+^ T cells (154). In addition, some studies have found that *Lactobacillus gallinarum* can promote the apoptosis of CRC cells by secreting a protective metabolite indole-3-lactic acid, thereby preventing the occurrence of CRC (155). *Lactobacillus reuteri* metabolizes to produce reuterin, which inhibits CRC growth by inducing oxidative stress and inhibiting protein translation (156). Surgical castration is one of the main methods for the treatment of prostate cancer (157). However, castration resistance after castration is an important reason for the development of prostate cancer (158). Pernigoni et al. (47) found that treatment with a combination of broad-spectrum antibiotics slowed the development of prostate cancer in mice. *Ruminococci* enriched in the gut microbiota of castration-resistant mice have the ability to convert pregnenolone and hydroxypregnenolone to downstream androgenic steroids (dehydroepiandrosterone [DHEA] and testosterone).

**Figure 2:**
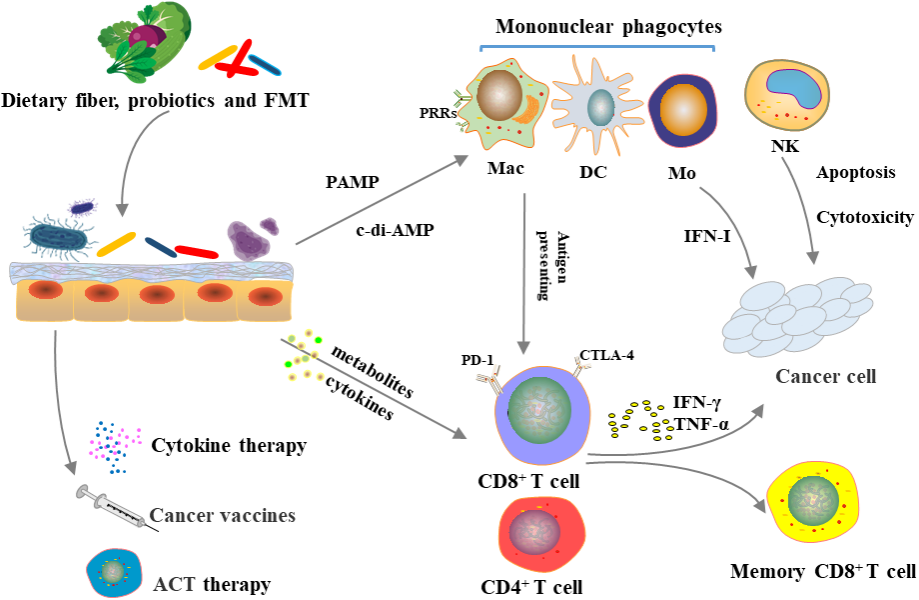
Microbiome, immune system and cancer immunotherapy.Diet, probiotic use and FMT can alter gut microbiota. The microbiota can directly influence innate and adaptive immunity or indirectly influence immune system through metabolism, which in turn affects the efficacy of immunosuppressive checkpoint inhibitors, cytokine therapy, tumor vaccines, and adoptive cell transfer therapy.

In general, the modification and application of microorganisms have great contribution to tumor immunotherapy, including improving efficacy and reducing side effects (**Figure 2**). At the same time, the microbiota, as a therapeutic target, also plays an important role in tumor treatment and prevention. However, microbial-targeted measures have largely focused on gut microbes, while applications to colonization of other epithelial barrier microbes remain to be expanded.

## Conclusion

The microbiota directly or indirectly activates and regulates the host’s immune system. Cancer immunotherapy, as a strategy that relies on the autoimmune system to fight tumors, has been proved to be related to the microbiota by several studies. In this review, we summarize the relationship between oncogenic microbial infection, microbiota dysbiosis, and carcinogenesis, and describe the relevant mechanisms. We also link the microbiota and tumor immunity from three perspectives (innate immunity, adaptive immunity, and metabolism). The impact of crosstalk between the microbiota and its metabolites on innate immune cells (NK, macrophages, and DCs) and effector T cells (especially CD8^+^T) on immunotherapy is described. Finally, we summarize the role of microbial modification and application in various tumor immunotherapies (ICIs, ACT, cytokine therapy, and tumor vaccines), including the use of microorganisms themselves as targets to treat and prevent cancer.

It is worth noting that the interaction of microbiota, immune system, and immunotherapy is complex; therefore, some problems still persist regarding the participation of microbiota in immunotherapy (4, 146). As discussed in a previous section, precise probiotic supplementation requires expanded basic research and preclinical trials. Besides, existing studies have shown that the use of probiotics in cancer immunotherapy is not necessarily positive, and some probiotics may hinder the effect of immunotherapy and may even promote cancer progression (132, 159). Therefore, better preparation should be done before conducting human trials to study the effect of commercially available probiotics on cancer immunotherapy. Although FMT may have an effect on ICIs, the effect of FMT on the reinduction of anti–PD-1 immunotherapy in patients with refractory metastatic melanoma is suboptimal, with only 30% of patients benefiting from it in one clinical trial (149). Furthermore, there are many side effects of FMT, such as abdominal discomfort, cramping, bloating, diarrhea, or constipation, which emphasizes higher FMT donor requirements (160). In addition, the composition of the microbiota varies in different individuals, and this is affected by multiple factors such as age, diet, circadian rhythm, as well as medication exposure (161). The uncertainty brought about by these factors also brings challenges for microorganisms in tumor immunotherapy. Therefore, future research may be able to combine multi-omics analysis, such as carefully characterizing the biological characteristics of microorganisms through genome sequencing and biochemical/microbiological analyses, to develop combinations of specific bacterial strains to treat various diseases including tumors (162).

Collectively, our review elucidates some of the mechanisms by which the microbiome contributes to cancer and cancer immunotherapy. These mechanisms also provide novel strategies for microbe-based cancer immunotherapy.

## Author contributions

Conceptualization: PZ and JL. Writing –original draft: PZ and XW. Writing –review & editing: PZ, YH, LS, and XL.Visualization: PZ, YH, JY, YJZ, FZ, YZ and HS. All authors contributed to the article and approved the submitted version.

## Funding

This work was supported by the National Natural Science Foundation of China (81472650, 81673061, 31271483, 81573050, 31872739, 30300313); National Science and Technology Major Project (2019ZX09201003-003, 2018ZX09733001-001-006, 2013ZX09301304001-003, 2012ZX10002006—003—001, 2009ZX09103-714); Sichuan Provincial Outstanding Youth Fund (2015JQO025); Key Research and Development Program of Sichuan Province (2020YFS0271); Applied Basic Research Program of Sichuan Province (2008SZ0093).

## Conflict of interest

Author HS was employed by company Sichuan Aupone Pharmaceutical Co., Ltd,.

The remaining authors declare that the research was conducted in the absence of any commercial or financial relationships that could be construed as a potential conflict of interest.

## Publisher’s note

All claims expressed in this article are solely those of the authors and do not necessarily represent those of their affiliated organizations, or those of the publisher, the editors and the reviewers. Any product that may be evaluated in this article, or claim that may be made by its manufacturer, is not guaranteed or endorsed by the publisher.
